# Flexible Terahertz Metamaterial Biosensor for Ultra-Sensitive Detection of Hepatitis B Viral DNA Based on the Metal-Enhanced Sandwich Assay

**DOI:** 10.3389/fbioe.2022.930800

**Published:** 2022-08-05

**Authors:** Yumin Li, Xiaojing Wang, Yu Liu, Weidong Jin, Huiyan Tian, Fengxin Xie, Ke Xia, Xiuming Zhang, Weiling Fu, Yang Zhang

**Affiliations:** ^1^ Medical Laboratory of the Third affiliated Hospital of Shenzhen University, Shenzhen, China; ^2^ Department of Laboratory Medicine, Southwest Hospital, Third Military Medical University (Army Medical University), Chongqing, China; ^3^ Department of Laboratory Medicine, Chifeng Municipal Hospital, Chifeng, China; ^4^ Department of Laboratory Medicine, Chongqing University Cancer Hospital, Chongqing, China

**Keywords:** terahertz metamaterials, biosensors, virus DNA, gold magnetic nanoparticles, clinical diagnosis

## Abstract

The high sensitivity and specificity of terahertz (THz) biosensing are both promising and challenging in DNA sample detection. This study produced and refined a flexible THz MM biosensor for ultrasensitive detection of HBV in clinical serum samples based on a gold magnetic nanoparticle-mediated rolling circle amplification (GMNPs@RCA) sandwich assay under isothermal conditions. Typically, solid-phase RCA reactions mediated by circular padlock probes (PLPs) are triggered under isothermal conditions in the presence of HBV DNA, resulting in long single-stranded DNA (ssDNA) with high fidelity and specificity. Then, the resultant ssDNA was conjugated with detection probes (DPs) immobilized on gold nanoparticles (DP@AuNPs) to form GMNPs-RCA-AuNPs sandwich complexes. The HBV DNA concentrations were quantified by introducing GMNPs-RCA-AuNPs complexes into the metasurface of a flexible THz metamaterial-based biosensor chip and resulting in a red shift of the resonance peak of the THz metamaterials. This biosensor can lead to highly specific and sensitive detection with one-base mismatch discrimination and a limit of detection (LOD) down to 1.27E + 02 IU/ml of HBV DNA from clinical serum samples. The HBV DNA concentration was linearly correlated with the frequency shift of the THz metamaterials within the range of 1.27E + 02∼1.27E + 07 IU/ml, illustrating the applicability and accuracy of our assay in real clinical samples. This strategy constitutes a promising THz sensing method to identify virus DNA. In the future, it is hoped it can assist with pathogen identification and clinical diagnosis.

## Graphical Abstract

Li et al. presented a new terahertz (THz) biosensing strategy for serum Hepatitis B virus DNA (HBV DNA) detection in clinical samples based on a flexible MM chip with an ultrathin polyethylene terephthalate (PET) substrate and gold magnetic nanoparticle-mediated rolling circle amplification (GMNPs@RCA) sandwich assay under isothermal conditions. This THz biosensing strategy demonstrates excellent analytical performance towards serum HBV DNA with high sensitivity, ultralow detection limit, excellent specificity and accuracy, as well as good stability, suggesting a promising THz sensing platform for virus DNA in clinical diagnosis, pathogen detection, and environmental monitoring in the future.

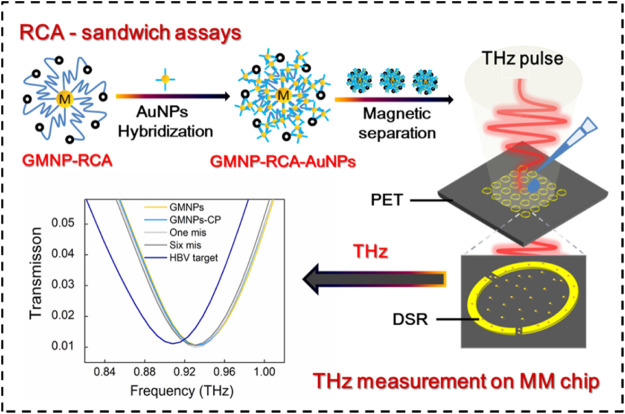

## Introduction

Virus infection remains a worldwide public health problem. Virus infections are diagnosed using traditional immunoassays to detect certain antigens and antibodies (Amini et al., 2017; Abusalah et al., 2020; Ong et al., 2021; Inoue et al., 2021). Alternatively, nucleic acid tests can be deployed to target a given genomic sequence. Virus nucleic acids are used as key markers of virus infection and replication. In addition, quantification of viral nucleic acids can be utilized in the early diagnosis, treatment, and assessment of how an individual is responding to antiviral therapy (Amini et al., 2017; Hsieh et al., 2022a). There is an urgent need to produce a diagnostic device, such as biosensing for infectious samples detection, which can carry out rapid, sensitive, and accurate detection of viral infection and replication during its early stages (Moitra et al., 2021; Hsieh et al., 2022b; Guo et al., 2022; Dighe et al., 2022; Liang et al., 2022; Moitra et al., 2022).

Terahertz (THz) spectroscopy has proven to have the potential to detect nucleic acids. THz spectroscopy utilizes forms of radiation that are positioned between microwave radiation and infrared radiation in the electromagnetic spectrum. The frequency range of the radiation used here is between 0.1 and 10 THz. Nucleic acids consist of nucleotide linear polymers joined together by phospholipid bonds. Nucleic acids’ THz spectrums can be used to determine the molecules’ configuration characteristics and their intermolecular collective and lattice vibrations. THz time-domain spectroscopy (TDS) has been readily applied to analyze nucleic acids in a label-free manner, such as in the identification of nucleotide bases (Fischer et al., 2002; Pickwell-MacPherson and Wallace, 2009; Wang et al., 2017), nucleic acid chain molecules (both single and double) (Bolivar et al., 2002), DNA mutations (Tang et al., 2015), transgenic genome (Yang et al., 2018), microcystin aptamer (Zhang et al., 2019), and even in the quantitative detection of DNA in aqueous solution (Arora et al., 2012; Hu et al., 2016; Yang et al., 2017; Zhou R. et al., 2021). However, there is an urgent need for new techniques and materials that are highly sensitive to THz-TDS for use in DNA detection.

Metamaterial (MM)-based THz biosensor chips have recently become a promising protein or DNA detection platform (Geng et al., 2017; Xu et al., 2017; Yang et al., 2018; Zhou R. et al., 2021). THz MM biosensor was designed as a planar array of metal double-split rings (DSRs) with asymmetric structures to obtain a tailored electromagnetic response, which is very sensitive to microenvironment medium change on the surface of the MM (Yang et al., 2018; Zhou J. et al., 2021; Zhou R. et al., 2021). These biosensors overcame the limit in sensitivity with typical THz-TDS systems, which might be attributed to the following factors: Firstly, asymmetric split resonators were designed to produce an exceptionally sharp resonance and achieve a great frequency shift of the THz transmission spectra. Secondly, low-permittivity materials were used to fabricate the substrates of the THz MM biosensor in an effort to minimize induced capacitance and transmission loss. Polyethylene terephthalate (PET) is an ideal flexible MM and a popular electro-optical substrate for various THz biosensor applications due to its low dispersion and loss in the THz range, high optical transparency, good surface smoothness, and simple fabrication of different thicknesses (Chen and Pickwell-MacPherson, 2019; Xia et al., 2019; Hu, et al., 2019; Zheng, et al., 2020; Wang, et al., 2021).

In addition, in DNA detection, THz-TDS sensitivity can be boosted through the utilization of signal amplification methods, including but not limited to rolling circle amplification (RCA) and polymerase chain reaction (PCR) (Arora et al., 2012; Yang et al., 2017). The target oligonucleotide hybridizes with the circular-shaped RCA template, which is a padlock probe (PLP), and then the long nucleic acid products with high fidelity are generated at a constant temperature (Hu and Zhang, 2010; Tong et al., 2012; Ding et al., 2013). Meanwhile, due to their unique physicochemical properties and large surface area, gold nanoparticles (AuNPs) are increasingly applied to develop RCA assays. AuNPs allow stable immobilization of oligonucleotide probes due to their adhesion to metal surfaces *via* thiol groups. RCA is then performed again following successful hybridization with the probes on the gold slide and the target sequence. Besides, AuNPs are also able to bind to RCA products; the effect of this is to enhance the plasmon resonance of incident light excitation whilst also elevating the transduction of small refractive index changes on the surface. Based on these considerations, the RCA combined with the AuNPs method has been subjected to a lot of attention for its use in the ultrasensitive detection of DNA, including AuNPs-RCA-based surface plasmon resonance (SPR) biosensors and RCA-surface enhanced Raman spectroscopy (SERS) sandwich assays (Shi et al., 2014; Guven et al., 2015).

AuNPs have also been introduced into THz MM biosensors. By integrating the THz plasmonic metasurface and AuNPs with each other, the metasensor’s sensitivity can be increased whilst also achieving a large resonance (Ahmadivand et al., 2018). However, when using RCA in combination with THz spectroscopy, the RCA products and complex matrix components (buffer) must be separated to minimize the background signal before THz measurements are taken (Yang et al., 2017). The use of gold magnetic nanoparticles (GMNPs) possesses not only the advantage of superparamagnetism to enable the isolation or extraction of target nucleic acids (Guven et al., 2015), but also the outstanding properties of AuNPs to enhance the capturing of targets in the surrounding medium of the THz MM and improve the sensitivity of the metasurface.

This research involved the fabrication of a THz flexible MM biosensor for the highly sensitive, highly selective determination of hepatitis B virus (HBV) DNA in clinical serum samples using a gold magnetic nanoparticle-mediated rolling circle amplification (GMNPs@RCA) sandwich assay under isothermal conditions. The flexible MM chip with a transparent and ultrathin PET substrate was designed as a planar array of metal double-split rings (DSRs) with asymmetric structures. The inherent properties of the excellent specificity of the circular PLP mediated specific target binding and the signal amplification of solid-phase RCA on GMNPs can maintain the high fidelity and sensitivity of this THz MM biosensor. In order to further enhance the detection signal, the AuNPs immobilized with the detection probes (DPs) are used to bind to the RCA products to form the GMNPs-RCA-AuNPs complex, which will achieve triple signal amplification of THz MM sensing. This THz biosensing strategy demonstrates excellent analytical performance in relation to HBV DNA with high sensitivity, ultra-low detection limits, excellent specificity and accuracy, as well as good stability. Together, these features indicate we have developed a promising THz sensing platform to identify viral DNA that can be used to carry out pathogen detection, clinical diagnosis, and environmental monitoring in the future.

## Experimental Methods

### Reagents and Materials

*E.coli* DNA ligase, Exonuclease I, Exonuclease III, dNTPs, DL2000 DNA Maker, and 50 bp DNA Ladder were bought from Takara Biomedical Tech Co., Ltd. (Dalian, China). The Qlysisi-V Kit was bought from Sangon Biotech Co., Ltd. (Shanghai, China). The Phi 29 DNA polymerase was obtained from Thermo Scientific (United States). Gold magnetic nanoparticles (GMNPs) with a diameter of 100 nm and gold nanoparticles (AuNPs) with a diameter of 40 nm were from XFNANO Materials Tech Co., Ltd. (Nanjing, China). Only analytical reagent grade chemicals are used in this study. Finally, a Milli-Q water purification system was used to prepare high-purity deionized water (Millipore Co., Bedford, United States).

### Oligonucleotides

All of the oligonucleotides used in this research were synthesized and purified by Takara Biotechnology Co., Ltd. (Dalian, China) through high-performance liquid chromatography (HPLC). The related sequences are listed in Table 1. The details of the HBV target sequence selection and its complementary padlock probe (PLP) design can be referred to in our previous report (Yao et al., 2013). PLP’s 5′ end was phosphorylated to achieve ligation. The 5′ end of the capture probe (CP) was altered by the addition of an SH group onto the GMNPs for immobilization. The 3′ end of the detection probe (DP) was also altered by the addition of an SH group onto the AuNPs for immobilization.

**TABLE 1:** Sequences of oligonucleotides used in the study.

Oligonucleotides	Sequences (5′-3′)
Padlock probe (PLP)	5′-Phosphate-TGCAGTTTCCGTCCGTAGTAGAATGAAGATAGC**GCATCGTAGGAGGACGGAGG**ATGATGGGTATGGGAATACAGG-3′
HBV target sequence	5′-CTACGGACGGAAACTGCACCTGTATTCCCATACCCATCAT-3′
Single-base mismatch	5′-CTA​CGG​ACG​GAA​ACT​GCA*A*CTG​TAT​TCC​CAT​ACC​CAT​CAT-3′
Six-base mismatch	5′-CTA​CGG​ACG​GAA​ACT​GC*GAG​CCG*ATT​CCC​ATA​CCC​ATC​AT-3′
Capture probe (CP)	5′-SH-TTTTTTTTTT**CCTCCGTCCTCCTACGATGC**-3′
Detection probe (DP)	5′-CCGTAGTAGAATGAAGATAGCGCATCG-SH-3′

The sequence underlined in the PLP matches the underlined sequence in the HBV target sequence, and the bold sequence in the PLP is complementary to the bold sequence in the capture probe (CP). A non-complementary sequence to the PLP was used as a control sequence, which included single-base mismatched and six-base mismatched sequences (italics).

### Gold Magnetic Nanoparticle-Rolling Circle Amplification-AuNPs Sandwich Assay Designs

The CP-coated gold magnetic nanoparticles (GMNPs-CP) were prepared. The 1 ml of GMNPs (0.05 mg/ml) was immobilized with 50 μl of 1% SDS and one OD CP by incubating for 20 min at room temperature to form an Au-S covalent bond. Then, 25 μl of 2 M NaCl was incorporated into the above mixture for 10 s in Ultrasonic. The mixture was then incubated at room temperature for 20 min. Subsequently, 25 μl of 2 M NaCl and 0.25 μl of 1% SDS were added to the above mixture for 10 s in ultrasonic, and the mixture was incubated for 20 min at room temperature. After repeating this process 20 times, the final solutions containing 0.9∼1.0 M NaCl and 0.01% SDS were incubated overnight at room temperature. Afterwards, the mixture was spun in a centrifuge to remove excess CPs. Finally, the GMNPs-CP were collected and resuspended in 0.01% SDS solutions.

**FIGURE 1:**
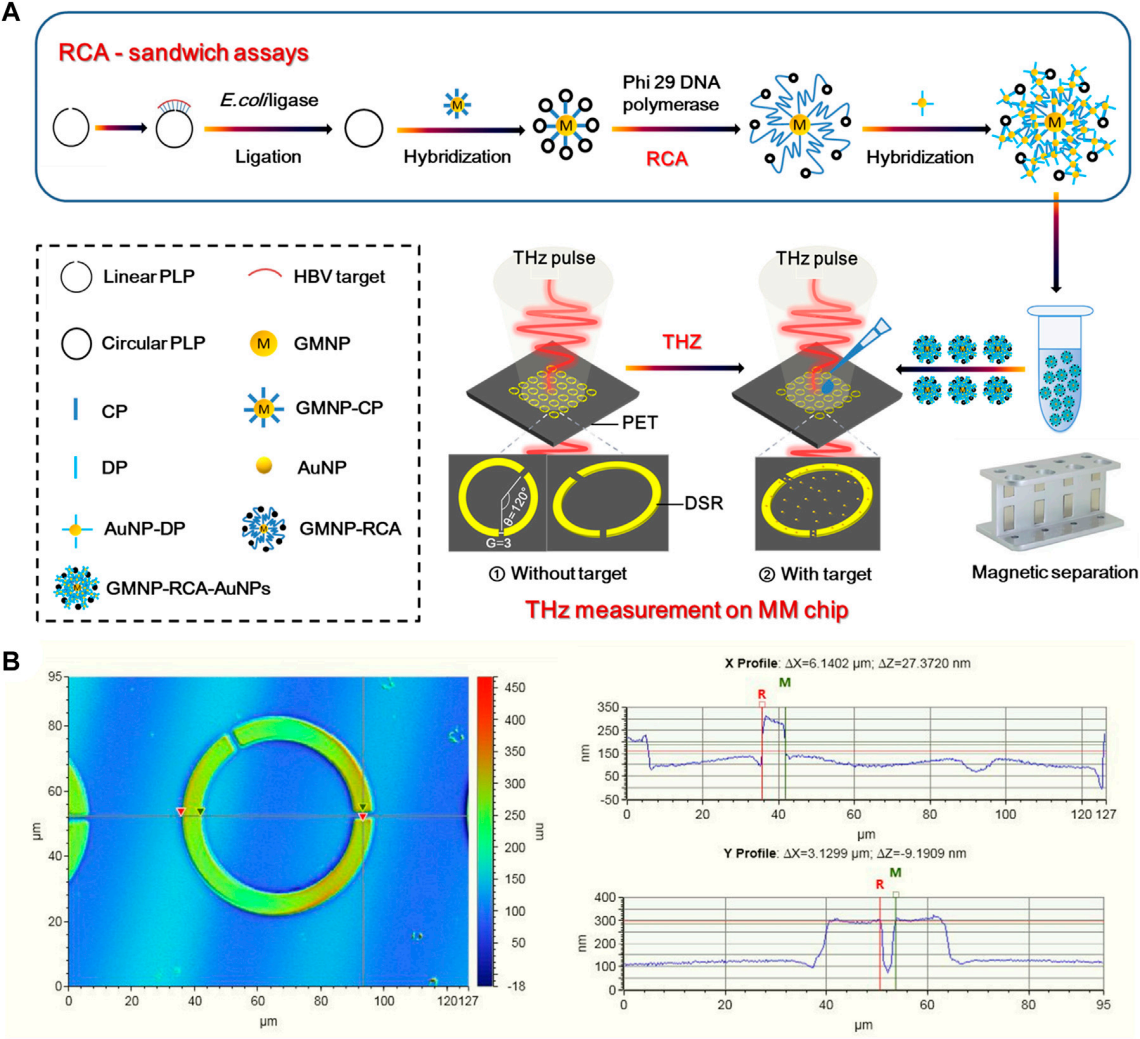
THz biosensing strategy for HBV DNA detection. **(A)** Schematic description of the THz MM biosensor chip for HBV DNA detection based on the GMNPs-RCA-AuNPs sandwich assay. **(B)** Characterization of DSR cell in a square lattice with dimensions of width and (G).

The schematic illustration of the stepwise assay design process of the solid-phase RCA reaction on GMNPs was shown in Figure 1A. At the first step to prepare the circular PLP, 17 μl of solutions containing 600 nM HBV target sequence, 100 nm linear PLP, and *E. coli* ligase buffer (30 mM Tris-HCl (pH 8.0), 4 mM MgCl_2_, 10 mM (NH_4_)_2_SO_4_, 1.2 mM EDTA, and 100 μM NAD) were denatured by heating them to 95°C for 5 min before cooling them to 4°C. Next, 10 U of *E. coli* ligase and 0.005% BSA were added to the mixture, which was then incubated at 16°C for 60 min. Subsequently, 10 U of each Exonuclease I and 200 U of Exonuclease III were incorporated into the ligation mixture at 37°C for 30 min. The purpose of this step was to remove any unligated PLPs or excess linear oligonucleotides in 40 μl of the reaction mixture [67 mM glycine-KOH (pH 9.5), 6.7 mM MgCl_2_, and 1 mM dithiothreitol (DTT)]. After holding at 95°C for 5 min, the reaction was terminated. After ligation and enzyme digestion reactions, 2 μl of GMNPs-CP and 20 μl of ligation and exonuclease products (circular PLPs) were introduced to microcentrifuge tubes at 37°C for 30 min to perform the first stage of hybridization between CP and circular PLPs. Once the hybridization step was completed, the GMNPs were washed with PBS buffer twice to detach any unhybridized oligonucleotides from the GMNPs’ surface. Next, 10 U of Phi 29 DNA polymerase and 2 μl of 100 μM dNTPs were introduced into the hybridization mixture at 40°C for 60 min to perform solid-phase RCA in 30 μl of the reaction mixture [33 mM of Tris-acetate (pH 7.9), 10 mM of Mg-acetate, 66 mM of K-acetate, 0.1% Tween 20, and 1 mM of DTT] (Yao et al., 2013). After the RCA step, PBS buffer was used to rinse the GMNPs with immobilized RCA products (GMNPs-RCA) twice.

AuNPs and GMNPs were used for the same purpose to prepare for probes coated with nanoparticles. Therefore, the same protocol was applied for AuNPs to prepare for DP-coated AuNPs (DP-AuNPs). At the second hybridization step, the GMNPs-RCA and DP-AuNPs were mixed in 50 μl of the reaction mixture (10 mM Tris, 1 mM EDTA, and 50 mM NaCl). After 15 min at 60°C, PBS was used to rinse the hybridization products (GMNPs-RCA-AuNPs complex) twice. They were then resuspended in the same volume of deionized water in preparation for subsequent THz measurements.

### Terahertz Metamaterial Chip Design and Fabrication

Figure 1A presents a diagram of a THz MM-based biosensor. To increase detection sensitivity, the designed MM metal split-ring (SR) resonators are composed of double splits with an asymmetric structure fabricated on an ultrathin PET film with a low intrinsic loss. The DSR sensor features strong sensing characteristics relating to the resonance shift loaded with a dielectric material (Geng et al., 2017; Yang et al., 2018). The THz MM chip consists of fundamental circuit elements. Moreover, its sensing mechanism is that the equivalent capacitance of the DSRs is sensitive to the change of environment refractive index. Notably, the equivalent capacitance changes when the sample on the DSRs-MM structure is altered; this then prompts the resonant frequency shift, which is an indicator of the sample’s presence. The period of DSR has a cell size of 90 μm^2^ × 90 μm^2^. As a DSR resonator, the central angle formed by the two radius crossing the center of two gaps was 120° (Figure 1A) and the gap (G) was 3 μm (Figure 1B), the width of the split ring was 6 μm (Figure 1B), and the inner radius and outer radius were 24 and 30 μm (Supplementary Figure S1), respectively. A 200-nm-thickness of gold was deposited on a PET sheet with thickness of 25 microns by radio frequency magnetron sputtering method. The main fabrication processes included lithography to form DSRs patterns, Au deposition, and lift-off. An image of the THz MM chip is shown in the supplementary information (Supplementary Figure S2).

### Extraction of Hepatitis B Virus DNA

The specimens were gathered from an HBV infected patient in Chifeng Municipal Hospital (Chifeng, China), which initially confirmed that the patient did not have the viruses that cause hepatitis A, C, D, E or human immunodeficiency virus (HIV) infections. The serum sample with HBV DNA concentration of 1.27E + 7 IU/ml was diluted to a concentration at 1.27E + 2 IU/ml with the negative serum, and then a series of samples with six equally spaced concentrations were provided for the sensitivity assay. HBV DNA was extracted from 200 μl of serum samples by following the procedure set out by the manufacturer, Qlysisi-V Kit, from Sangon Biotech Co., Ltd. (Shanghai, China). The aforementioned RCA protocol was used to detect HBV DNA extractives.

### Terahertz Spectroscopy Measurement

In the present study, THz spectroscopic measurements were carried out using a commercial THz-TDS system set to transmission mode (TAS7500SP, Advantest). The measured frequency range was from 0.1 to 2 THz with a frequency resolution of 7.6 GHz. The transmission measurements were carried out using linearly polarized THz waves at normal incidence, with the electric field positioned parallel to the gap. The samples were measured in a dry nitrogen atmosphere at 25°C. A blank PET sheet identical to the array substrate was employed to derive the THz reference pulse. The THz MM chip was used as the THz detection pulse, both with and without the target (Figure 1A). Once the THz measurements were taken, a Fourier transformation was applied to obtain the frequency domain data from the time-domain. A frequency shift was calculated by *Δf* = *f*_*target*_–*f*_without_ _*target,*_ where *f*_*target*_ represents the resonance peak frequency of the samples, and *f*_without_ _*target*_ symbolizes the resonance peak frequency of the bare THz metamaterial chip without samples.

## Results and Discussion

### Principle of Terahertz Biosensing Strategy for Hepatitis B Virus DNA Detection

Three steps were demonstrated in the THz biosensing strategy (Figure 1A). Firstly, the solid-phase RCA reaction on GMNPs was performed to realize the first detection signal amplification of the target sequence, and then the AuNPs immobilized with the DPs were used to bind to the RCA products to form the GMNPs-RCA-AuNPs complex, which further realizes the second detection signal amplification of RCA products. Secondly, the THz MM chip with a transparent and ultrathin PET substrate was designed as a planar array of gold DSRs with asymmetric structures. This chip can sense significant shifts in the resonance frequency caused by changes in the metasurface’s dielectric environment. Thirdly, by introducing the GMNPs-RCA-AuNPs complex to the THz MM chip, the target DNA was detected by THz measurements with high sensitivity and accuracy, which will realize the third detection signal amplification. Therefore, this strategy will achieve triple signal amplification of THz MM sensing.

### Feasibility of Terahertz Metamaterial Biosensing Based on Gold Magnetic Nanoparticle-Mediated Rolling Circle Amplification

**FIGURE 2:**
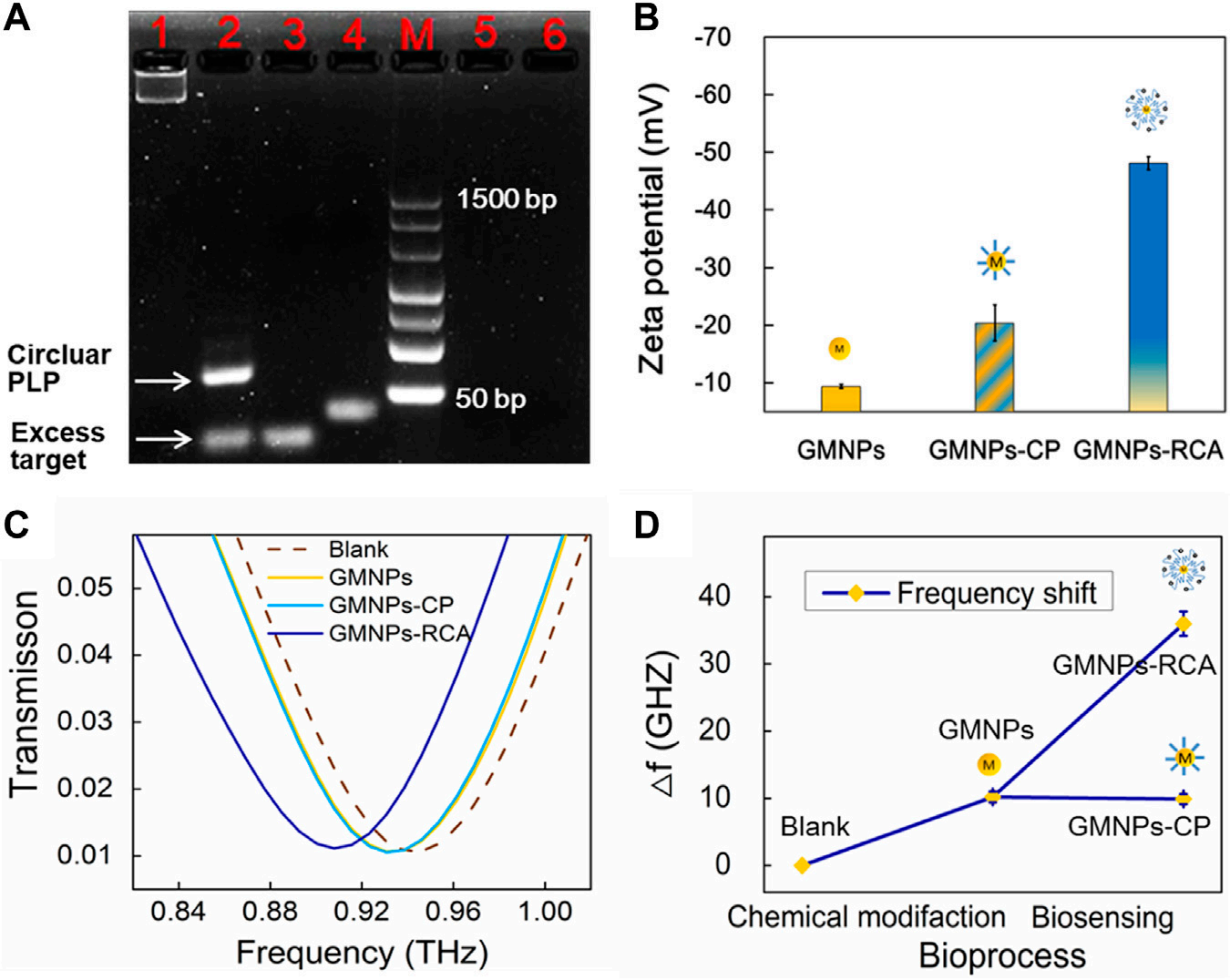
GMNPs-RCA assay for HBV target sequence. **(A)** Electrophoretic identification of the ligation reaction mixture and RCA products. Lanes 1, 2, 3, 4, 5, and 6 represent the RCA products, ligation products (circular PLP), target, linear PLP, single-base mismatched sequences, and six-base mismatched sequences, respectively. **(B)** Zeta potential of GMNPs, GMNPs-CP, and GMNPs-RCA. Error bars indicate the SD (*n* = 3). **(C)** THz measurements of the bare THz MM chip (Blank), GMNPs, GMNPs-CP, and GMNPs-RCA. **(D)** The corresponding THz frequency shifts (*Δf*) of Figure 2C. Error bars indicate the SD (*n* = 3). *Δf* = *f*_*target*_–*f*_without target_ of which *f* represents the resonance peak frequency.

3% agarose gel electrophoresis was used to assess the RCA reaction performance of Ligation and RCA products. Theoretically, when the linear PLP is hybridized with a mismatched strand, neither a specific reaction of circularization nor a series of amplification reactions are expected to occur in the follow-up process. As shown in Figure 2A, the PLPs could not hybridize with the single-base and six-base mismatched sequences. Additionally, as the subsequent exonuclease treatment removed any non-circularized PLPs, no amplification template remained to conduct RCA through agarose gel electrophoresis analysis. The circular PLP migrated at a slower rate than the linear PLP and the target sequence. The RCA products could not enter the gels because they were too large. Furthermore, zeta potential was used to validate the RCA reaction that occurred on the GMNP surface. This validation method was selected due to the negative charge on the ssDNA; specifically, the conjugation of GMNPs with ssDNA should give rise to negative charged conjugates that will render the zeta potential negative. As shown in Figure 2B, there was an observable change in the non-modified GMNPs’ value (−9.37 ± 0.37 mV) for the GMNPs-CP (−20.40 ± 3.15 mV). This shift indicates that the CPs were appropriately connected with the GMNPs. Following amplification, a marked shift to −48.13 ± 1.08 mV (Figure 2B) was recorded. This indicates that the GMNPs were covered with amplified DNA that had a greater negative charge.

The DSRs MM chips were used to detect different GMNPs by THz spectroscopy measurements. It should be noted that the asymmetric structure of DSRs had two dips. The low-frequency resonance peak (dip 1) and high-frequency resonance peak (dip 2) are at the sites of 0.259 and 0.946 THz (Supplementary Figure S3). The different GMNP suspensions were evaporated at 42°C in an oven. This ensured the DNA was not denatured and could later be used for THz spectroscopy measurements. Figure 2C shows the measured transmission spectra of dip 2 with the bare THz MM chip (blank), GMNPs, GMNPs-CP, and GMNPs-RCA (HBV target). The difference in the frequency of transmission spectra between GMNPs and GMNPs-CP was negligible, whereas the readily apparent difference between GMNPs and GMNPs-RCA was recorded due to the amplified DNA products on the GMNPs’ surfaces. The frequency shift was 25.8 GHz before and after the RCA procedure (Figure 2D). The results demonstrated that the gold magnetic nanoparticle-mediated RCA products could be sensitively captured on the THz MM chip surface for biosensing.

### Enhancement of Terahertz Sensitivity Using Gold Nanoparticles

**FIGURE 3:**
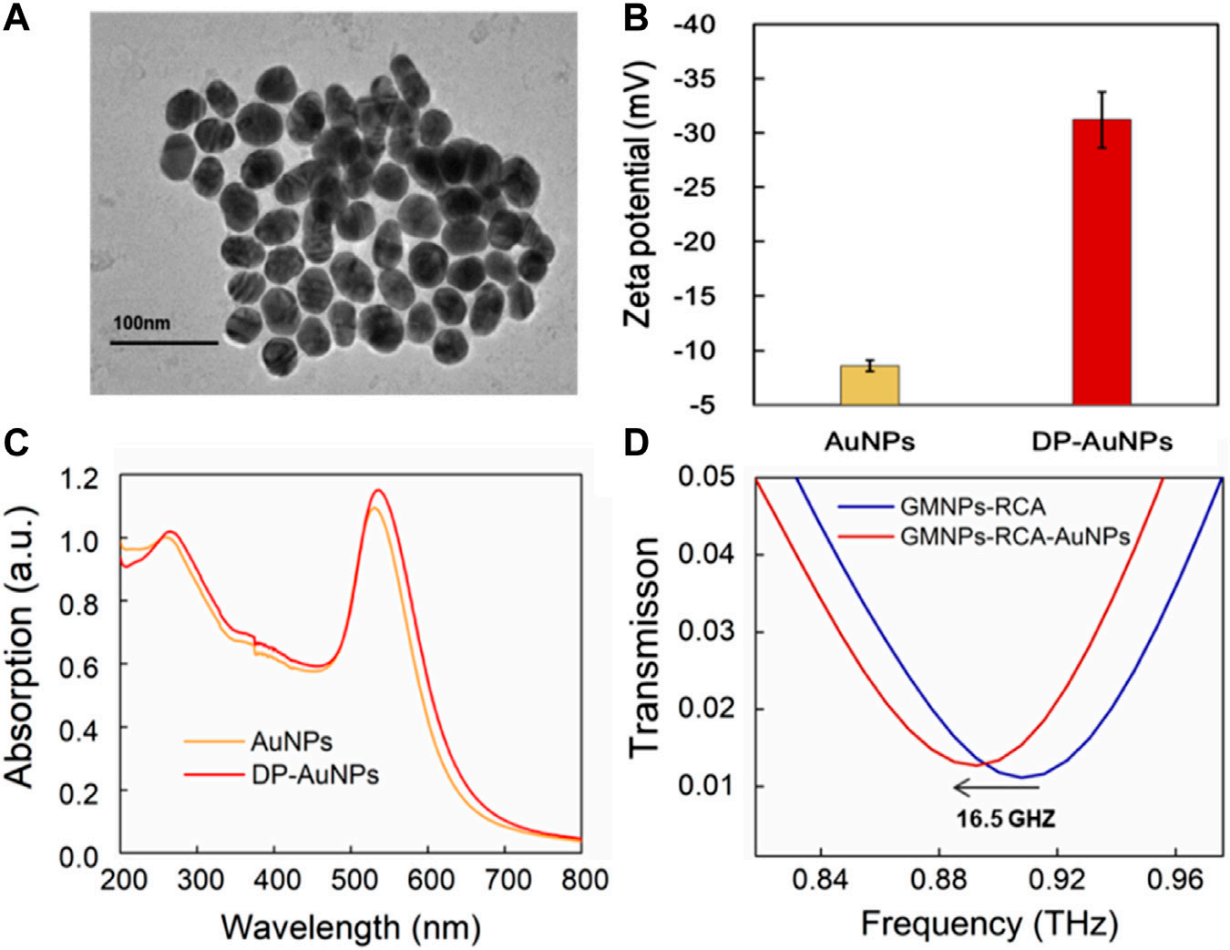
THz measurement for the GMNPs-RCA-AuNPs complex. **(A)** The TEM images of the AuNPs. **(B)** The zeta potential of AuNPs and DP-AuNPs. Error bars indicate the SD (*n* = 3). **(C)** The UV-Visible absorbance spectra of the AuNPs (Yellow) and the DP-AuNPs (Red). **(D)** The frequency shift of THz transmission spectra for GMNPs-RCA-AuNPs compared to GMNPs-RCA.

The localized electric field present in the gaps of the MM chip enhanced the interactions between the DNA and THz wave, which was further enhanced by the gold nanoparticles (AuNPs) due to their high refractive index, thus verifying that AuNPs can increase the THz biosensing signal (Yang et al., 2021; Zhan et al., 2021). Here, the GMNPs-RCA-AuNPs sandwich assay was refined to improve the sensitivity of DNA detection based on the THz MM biosensor. The impact of AuNP diameter on metamaterial sensing was explored to better understand the role it plays in signal enhancement, relative to the GMNPs-RCA method. AuNPs with an average diameter of 40 nm assume an almost round shape and exhibit good monodispersity, as shown in the TEM images (Figure 3A). They also undergo a shift to more negative values, as was observed in the zeta potential of AuNPs (−8.60 ± 0.53 mV) when the coverage by DP (−31.20 ± 2.59 mV) was higher (Figure 3B). Moreover, the AuNPs have a plasmon resonance at 531 nm (black line); meanwhile, the DP-modified AuNPs underwent a red shift from 531 to 536 nm (red curve, respectively), demonstrating that functionalization of the AuNPs for DP was successfully completed (Figure 3C). Then, it was obvious to see that the GMNPs-RCA-AuNPs complexes could be readily determined amongst GMNPs-RCA due to their higher THz frequency shift signals. There was a further frequency shift of transmission spectra at 16.5 GHz (Figure 3D) before and after the formation of the GMNPs-RCA-AuNPs complexes, exhibiting the larger magnitude of target DNA detection signal amplification by the THz biosensing strategy based on the GMNPs-RCA-AuNPs sandwich assay and MM chip, compared to the traditional RCA-THz MM biosensor method, which may be due to the gold-mediated nanoparticles’ (including GMNPs and AuNPs) high refractive index.

### Sensitivity of Terahertz Biosensing Strategy for Hepatitis B Virus Target Sequence

**FIGURE 4:**
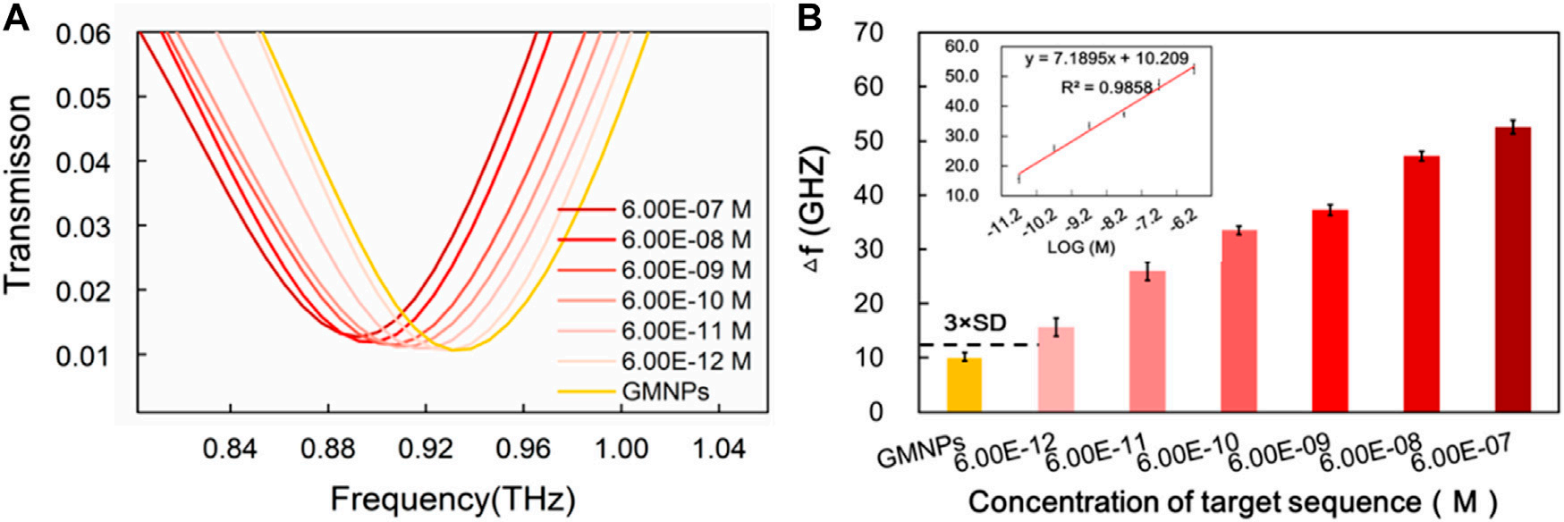
Sensitivity of THz biosensing strategy for HBV target sequence detection. **(A)** The frequencies of the THz transmission spectra for blank GMNPs and GMNPs-RCA-AuNPs amplified with different concentrations of the HBV target sequence. **(B)** The THz frequencies shifts (*Δf*) for blank GMNPs and GMNPs-RCA-AuNPs amplified with different concentrations of the HBV target sequence. Error bars indicate the SD (*n* = 3). The inset graph shows the linear fit of the frequencies (Δf) versus the logarithm of the HBV target sequence concentration. The error bars indicate the SD (*n* = 3).

The frequencies of the THz transmission spectra for various synthetic HBV target sequence concentrations are shown in Figure 4A. Improved frequency shift was remarkably observed with the increase of the HBV target sequence. The dose-dependent manner was further explored upon the addition of different concentrations of the HBV target. As it was producing a signal equal to the blank GMNPs signal plus three times its standard deviation (SD), a detection limit for the HBV target was estimated to be 6.00E-12M (Figure 4B), indicating that 6.00E-12M of the target sequence can be effectively detected. As can be seen from Figure 4B, the frequency and the logarithm of the HBV target sequence exhibited a notable linear correlation (*y* = 7.1895x + 10.209, R^2^ = 0.9858) at concentrations in the range of 6.00E-12∼6.00E-07M.

Compared to the reported RCA-based methods, the current assay’s sensitivity (6.00E-12M) is inferior to the AuNPs-RCA-based SPR biosensor assay (5.00E-13M) and the AuNPs-RCA-SERS assay (1.00E-13M) (Shi et al., 2014; Guven et al., 2015), but it is comparable with the RCA-SPR biosensor assay (5.00E-12M) and greatly exceeded our previous THz measurements for bacterial DNA using magnetic bead-based RCA (1.00E-10M) (Xiang et al., 2013; Yang et al., 2017). There are several factors that could produce the high sensitivity of the assay. Firstly, the circular DSR resonators made of double splits were specifically designed to produce highly sensitive THz MM chips. Secondly, the RCA produces large DNA sequences, which are conjugated on the GNMPs to improve the sensitivity of the THz MM chips. Thirdly, it is possible for a significant quantity of AuNPs to hybridize with the ssDNA products, which include tandem repeats numbering in the thousands, thereby further enhancing the sensitivity of THz MM chips. Moreover, magnetic separation of RCA products, extracts any interferences from the complex sample matrix when conducting THz measurements, which presents a time-saving replacement and allows the flexibility of applying DNA end point detection methods. Thus, the results of the present study suggest that a specific oligonucleotide sequence can be detected in a more sensitive and selective manner using the current assay compared to similar reported methods of DNA detection under THz spectroscopy conditions (Arora et al., 2012; Yang et al., 2017).

### Sensitivity of Terahertz Biosensing Strategy for Hepatitis B Virus DNA in Serum Sample

**FIGURE 5:**
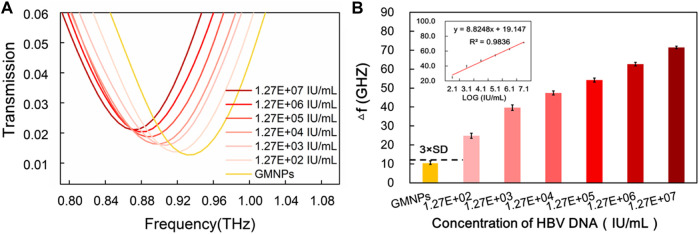
Sensitivity of THz biosensing strategy for HBV DNA detection in serum sample. **(A)** The frequencies of THz transmission spectra for blank GMNPs and GMNPs-RCA-AuNPs amplified with different concentrations of the HBV DNA. **(B)** The THz frequencies shifts (*Δf*) for blank GMNPs and GMNPs-RCA-AuNPs amplified with different concentrations of the HBV DNA. Error bars indicate the SD (*n* = 3). The inset graph shows the linear fit of the frequencies versus the logarithm of the HBV DNA concentration. The error bars indicate the SD (*n* = 3).

To further investigate the applicability of the proposed assay in a real biological environment, serum samples were selected to detect HBV DNA. As shown in Supplementary Figure S4, analysis of HBV DNA was performed on a 1% agarose gel after the RCA reaction. To assess the extent of this method’s sensitivity, serially diluted serum samples were measured (Figure 5A). Much like with the synthetic target sequence, THz transmission spectra’s analytical frequencies also exhibited a good linear relationship with the logarithm of the HBV DNA at concentrations in the range of 1.27E + 02∼1.27E + 07 IU/ml (*y* = 8.8248x + 19.147, R^2^ = 0.9836) range (Figure 5B). For the proposed strategy, the HBV DNA detection limit was estimated to be 1.27E + 02 IU/ml. This provides for a signal that is larger than the blank GMNPs signal plus three times its SD (Figure 5B).

The sensitivity of the current assay (1.27E + 02 IU/ml) is similar or superior to existing HBV detection methods reported elsewhere, including the loop-mediated isothermal amplification (LAMP) assay (100 IU/ml) (Akram et al., 2018), nanostructured impedance biosensor assay (1,000 copies/ml) (Chen et al., 2016), and the RCA-based quartz crystal microbalance (QCM) biosensor assay (10,000 copies/ml) (Yao et al., 2013). The experiment results suggested that THz spectroscopy measurements of HBV DNA by GMNPs-RCA-AuNPs sandwich assay integrated with the MM biosensor chip provided a new alternative method for early diagnosis and treatment of HBV. A recent study revealed that the highly efficient electrochemiluminescence (ECL) biosensor could realize the ultrasensitive detection of HBV DNA from 100 aM to 1 nM while the limit of detection was 18.08 aM (Guo et al., 2022). The more suitable metamaterials and better signal amplification strategies would be considered to further improve sensitivity of THz biosensor in the future study.

### Specificity of the Terahertz Biosensing Strategy

**FIGURE 6:**
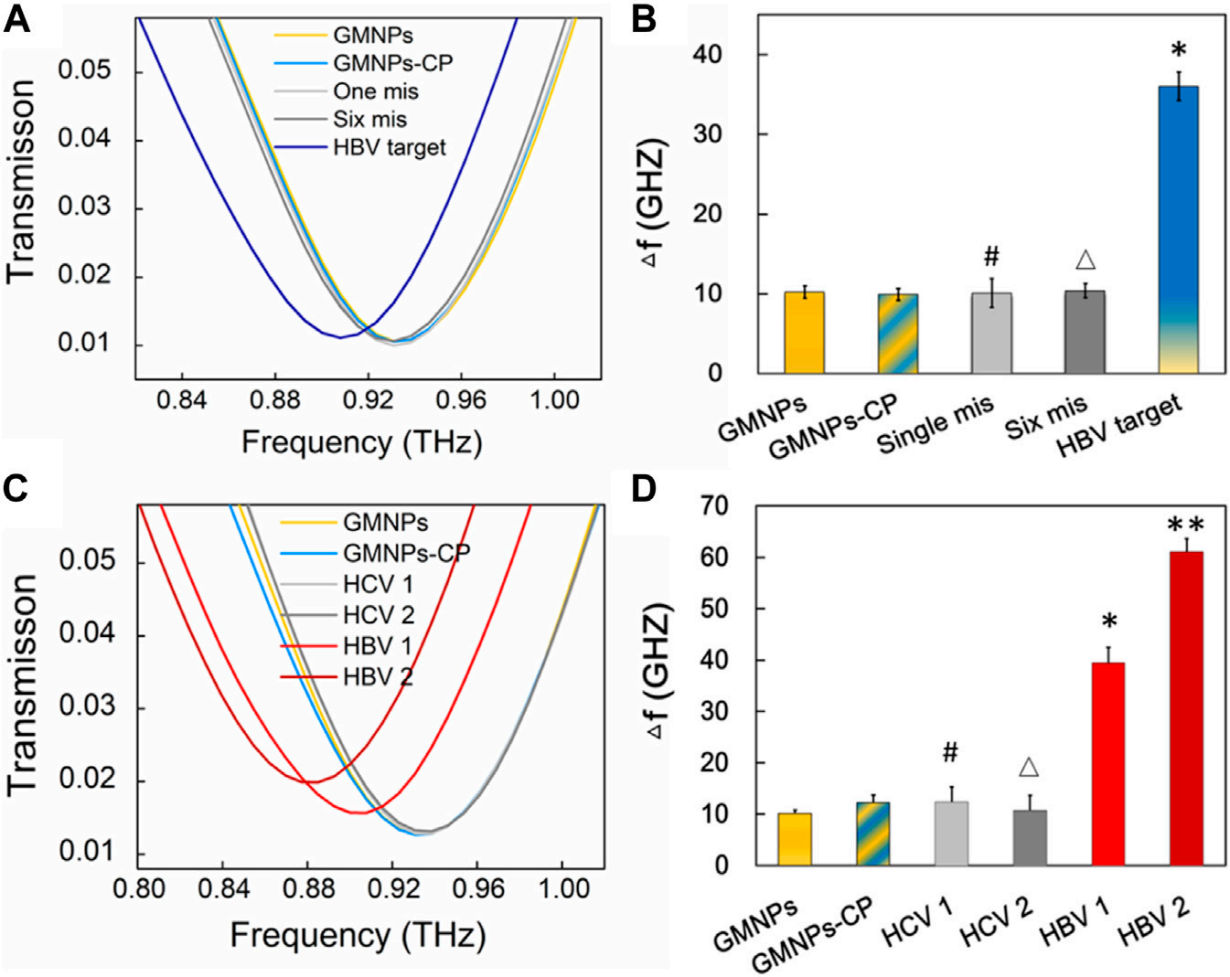
Specificity verification of the THz biosensing strategy. **(A)** THz transmission spectra for HBV target and mismatched sequence. **(B)** The frequency shifts (*Δf*) of THz transmission spectra. Error bars indicate the SD (*n* = 3). Significance was determined using one-way ANOVA; *p < 0.05, HBV target versus GMNPs; ^*#*^ *p* > 0.05, single-base mismatched sequence versus GMNPs; and ^*△*^ *p* > 0.05, six-base mismatched sequence versus GMNPs. **(C)** THz transmission spectra for HBV and HCV in serum samples. **(D)** The frequency shifts (*Δf*) of THz transmission spectra. Error bars indicate the SD (*n* = 3). Significance was determined using one-way ANOVA, *p < 0.05, HBV 1 (1.74E + 03 IU/ml) versus GMNPs, **p < 0.05, HBV 2 (1.58E + 06 IU/ml) versus GMNPs, ^*#*^ *p* > 0.05, HCV 1 (4.03E + 03 IU/ml) versus GMNPs, and ^*△*^ *p* > 0.05, HCV 2 (2.45E + 06 IU/ml) versus GMNPs.

In order to assess the specificity of the THz biosensing strategy for HBV DNA detection, the frequency shifts brought about by the HBV target sequence were compared with the shifts stemming from single-base and six-base mismatched oligonucleotides under the same detection conditions. The THz signals (Figure 6A) were measured along with GMNPs, GMNPs-CP, HBV target, single-base mismatched, and six-base mismatched sequences. As shown in Figure 6B, the Δ*f* of the HBV target was significantly greater than that of blank GMNPs (*p <* 0.05), GMNPs-CP, single-base mismatched and six-base mismatched sequences, respectively. The results demonstrated that the GMNPs-RCA reaction was highly selective for single-base discrimination. Moreover, the long ssDNA strands could be produced in a specificity sequence-dependent manner which enjoyed high fidelity. Additionally, no amplified frequency signal could be detected in the presence of mismatched sequences. Further detection results of clinical serum samples (Figures 6C,D) revealed that the HBV DNA in serum induced significantly greater frequency shifts (*Δf*) than blank GMNPs (*p* < 0.05), respectively. However, it is not significantly different between GMNPs and Hepatitis C (HCV) as the RCA products were absent (*p* > 0.05), respectively. The results suggest that the THz biosensing strategy developed here enjoys high selectivity and specificity for HBV DNA detection.

### Clinical Application of the Terahertz Biosensing Strategy and Reproducibility

A recovery experiment was performed by detecting six clinical human serum samples with different concentrations of HBV DNA over the linear range to assess the proposed THz biosensing strategy’s analytical reliability. The recovery rate was calculated (Table 2), the results of which aligned with the spiked amounts for the targets. This suggests that the measured and actual values in the serum sample are consistent. To evaluate the THz biosensing strategy’s reproducibility, the relative standard deviation (RSD) was calculated based on the four replicate measurements of the clinical human serum samples. The RSDs (%) were determined to be 2.94%–4.47%, indicating that the reproducibility is acceptable. The results showed that the proposed method exhibits sound precision and recovery when applied to clinical serum samples. It is recommended that clinical transformation should be further explored in future research.

**TABLE 2:** Determination results of HBV DNA in clinical serum samples (*n* = 4).

Samples	Added	Measured	RSD (%)	Recovery (%)
1	5.32E + 02 IU/ml	5.13E + 02 IU/ml	3.99	99.1
2	1.74E + 03 IU/ml	1.90E + 03 IU/ml	2.94	101.0
3	1.79E + 04 IU/ml	2.73E + 04 IU/ml	3.97	103.7
4	1.50E + 05 IU/ml	2.28E + 05 IU/ml	4.47	102.7
5	1.58E + 06 IU/ml	1.38E + 06 IU/ml	3.51	98.4
6	1.27E + 07 IU/ml	2.80E + 07 IU/ml	3.91	104.0

## Conclusion

This research developed a new assay for THz spectroscopy measurement of HBV DNA by using the combination of GMNPs-RCA-AuNPs sandwich assay and the THz MM biosensor chip. Leveraging the high amplification efficiency, specificity, and fidelity of RCA under isothermal conditions, as well as the intrinsically high sensitivity of the gold-mediated nanoparticles and THz MM biosensor chip, as low as 1.27E + 02 IU/ml of serum HBV DNA was detected by THz spectroscopy. The results also indicate there is a linear relationship between THz transmission spectra frequencies and HBV DNA logarithm at concentrations in excess of the 1.27E + 02∼1.27E + 07 IU/ml range. It is, therefore, pointed out that the proposed THz biosensing strategy can be employed to carry out the specific, sensitive, selective, and quantitative detection of virus DNA in clinical samples.

## Data Availability

The original contributions presented in the study are included in the article/Supplementary Material; further inquiries can be directed to the corresponding authors.

## References

[B1] Abusalah M. A. H. Gan S. H. Al-Hatamleh M. A. I. Irekeola A. A. Shueb R. H. Yean Yean C. (2020). Recent Advances in Diagnostic Approaches for Epstein-Barr Virus. Pathogens 9 (3), 226. 10.3390/pathogens9030226 PMC7157745 32197545

[B2] Ahmadivand A. Gerislioglu B. Tomitaka A. Manickam P. Kaushik A. Bhansali S. (2018). Extreme Sensitive Metasensor for Targeted Biomarkers Identification Using Colloidal Nanoparticles-Integrated Plasmonic Unit Cells. Biomed. Opt. Express 9 (2), 373–386. 10.1364/boe.9.000373 29552379 PMC5854044

[B3] Akram A. Islam S. M. R. Munshi S. U. Tabassum S. (2018). Detection of Hepatitis B Virus DNA Among Chronic and Potential Occult HBV Patients in Resource-Limited Settings by Loop-Mediated Isothermal Amplification Assay. J. Viral Hepat. 25 (11), 1306–1311. 10.1111/jvh.12931 29768691

[B4] Amini A. Varsaneux O. Kelly H. Tang W. Chen W. Boeras D. I. (2017). Diagnostic Accuracy of Tests to Detect Hepatitis B Surface Antigen: A Systematic Review of the Literature and Meta-Analysis. BMC Infect. Dis. 17 (1), 698. 10.1186/s12879-017-2772-3 29143619 PMC5688498

[B5] Arora A. Luong T. Q. Krüger M. Kim Y. J. Nam C.-H. Manz A. (2012). Terahertz-time Domain Spectroscopy for the Detection of PCR Amplified DNA in Aqueous Solution. Analyst 137 (3), 575–579. 10.1039/c2an15820e 22189821

[B6] Bolivar P. H. Brucherseifer M. Nagel M. Kurz H. Bosserhoff A. Buttner R. (2002). Label-free Probing of Genes by Time-Domain Terahertz Sensing. Phys. Med. Biol. 47, 3815–3821. 10.1088/0031-9155/47/21/320 12452572

[B7] Chen C.-C. Lai Z.-L. Wang G.-J. Wu C.-Y. (2016). Polymerase Chain Reaction-free Detection of Hepatitis B Virus DNA Using a Nanostructured Impedance Biosensor. Biosens. Bioelectron. 77, 603–608. 10.1016/j.bios.2015.10.028 26479905

[B8] Chen X. Pickwell-MacPherson E. (2019). A Sensitive and Versatile Thickness Determination Method Based on Non-inflection Terahertz Property Fitting. Sensors 19 (19), 4118. 10.3390/s19194118 PMC6806189 31547626

[B9] Dighe K. Moitra P. Alafeef M. Gunaseelan N. Pan D. (2022). A Rapid RNA Extraction-free Lateral Flow Assay for Molecular Point-Of-Care Detection of SARS-CoV-2 Augmented by Chemical Probes. Biosens. Bioelectron. 200, 113900. 10.1016/j.bios.2021.113900 34959185 PMC8684225

[B10] Ding C. Wang N. Zhang J. Wang Z. (2013). Rolling Circle Amplification Combined with Nanoparticle Aggregates for Highly Sensitive Identification of DNA and Cancercells. Biosens. Bioelectron. 42, 486–491. 10.1016/j.bios.2012.10.015 23238323

[B11] Fischer B. M. Walther M. Jepsen P. U. (2002). Far-infrared Vibrational Modes of DNA Components Studied by Terahertz Time-Domain Spectroscopy. Phys. Med. Biol. 47, 3807–3814. 10.1088/0031-9155/47/21/319 12452571

[B12] Geng Z. Zhang X. Fan Z. Lv X. Chen H. (2017). A Route to Terahertz Metamaterial Biosensor Integrated with Microfluidics for Liver Cancer Biomarker Testing in Early Stage. Sci. Rep. 7 (1), 16378–16411. 10.1038/s41598-017-16762-y 29180650 PMC5704020

[B13] Guo Y.-Z. Liu J.-L. Chen Y.-F. Chai Y.-Q. Li Z.-H. Yuan R. (2022). Boron and Nitrogen-Codoped Carbon Dots as Highly Efficient Electrochemiluminescence Emitters for Ultrasensitive Detection of Hepatitis B Virus DNA. Anal. Chem. 94 (21), 7601–7608. 10.1021/acs.analchem.2c00763 35575687

[B14] Guven B. Boyaci I. H. Tamer U. Acar-Soykut E. Dogan U. (2015). Development of Rolling Circle Amplification Based Surface-Enhanced Raman Spectroscopy Method for 35S Promoter Gene Detection. Talanta 136, 68–74. 10.1016/j.talanta.2014.11.051 25702987

[B15] Hsieh H.-Y. Luo J.-X. Shen Y.-H. Lo S.-C. Hsu Y.-C. Tahara H. (2022a). A Nanofluidic Preconcentrator Integrated with an Aluminum-Based Nanoplasmonic Sensor for Epstein-Barr Virus Detection. Sensors Actuators B Chem. 355, 131327. 10.1016/j.snb.2021.131327

[B16] Hsieh H.-Y. Chang R. Huang Y.-Y. Juan P.-H. Tahara H. Lee K.-Y. (2022b). Continuous Polymerase Chain Reaction Microfluidics Integrated with a Gold-Capped Nanoslit Sensing Chip for Epstein-Barr Virus Detection. Biosens. Bioelectron. 195, 113672. 10.1016/j.bios.2021.113672 34601264

[B17] Hu J. Zhang C.-y. (2010). Sensitive Detection of Nucleic Acids with Rolling Circle Amplification and Surface-Enhanced Raman Scattering Spectroscopy. Anal. Chem. 82, 8991–8997. 10.1021/ac1019599 20919697

[B18] Hu X. Xu G. Wen L. Wang H. Zhao Y. Zhang Y. (2016). Metamaterial Absorber Integrated Microfluidic Terahertz Sensors. Laser & Photonics Rev. 10, 962–969. 10.1002/lpor.201600064

[B19] Hu J. Lang T. Xu W. Liu J. Hong Z. (2019). Experimental Demonstration of Electromagnetically Induced Transparency in a Conductively Coupled Flexible Metamaterial with Cheap Aluminum Foil. Nanoscale Res. Lett. 14, 359. 10.1186/s11671-019-3180-y 31792628 PMC6888790

[B20] Inoue T. Matsui T. Tanaka Y. (2021). Novel Strategies for the Early Diagnosis of Hepatitis B Virus Reactivation. Hepatology Res. 51 (10), 1033–1043. 10.1111/hepr.13699 34272919

[B22] Liang L. Jiang Y. J. Zhang L. C. Liu H. Li Y. F. Li C. M. (2022). Rational Fabrication of a DNA Walking Nanomachine on Graphene Oxide Surface for Fluorescent Bioassay. Biosens. Bioelectron. 211, 114349. 10.1016/j.bios.2022.114349 35576722

[B23] Moitra P. Alafeef M. Dighe K. Sheffield Z. Dahal D. Pan D. (2021). Synthesis and Characterisation of N-Gene Targeted NIR-II Fluorescent Probe for Selective Localisation of SARS-CoV-2. Chem. Commun. 57 (51), 6229–6232. 10.1039/d1cc01410b 34048518

[B24] Moitra P. Chaichi A. Abid Hasan S. M. Dighe K. Alafeef M. Prasad A. (2022). Probing the Mutation Independent Interaction of DNA Probes with SARS-CoV-2 Variants through a Combination of Surface-Enhanced Raman Scattering and Machine Learning. Biosens. Bioelectron. 208, 114200. 10.1016/j.bios.2022.114200 35367703 PMC8938299

[B25] Ong D. S. Y. Fragkou P. C. Schweitzer V. A. Chemaly R. F. Moschopoulos C. D. Skevaki C. (2021). How to Interpret and Use COVID-19 Serology and Immunology Tests. Clin. Microbiol. Infect. 27 (7), 981–986. 10.1016/j.cmi.2021.05.001 33975005 PMC8106522

[B26] Pickwell-MacPherson E. Wallace V. P. (2009). Terahertz Pulsed Imaging-A Potential Medical Imaging Modality? Photodiagnosis Photodyn. Ther. 6, 128–134. 10.1016/j.pdpdt.2009.07.002 19683214

[B28] Shi D. Huang J. Chuai Z. Chen D. Zhu X. Wang H. (2014). Isothermal and Rapid Detection of Pathogenic Microorganisms Using a Nano-Rolling Circle Amplification-Surface Plasmon Resonance Biosensor. Biosens. Bioelectron. 62, 280–287. 10.1016/j.bios.2014.06.066 25022511

[B29] Tang M. Huang Q. Wei D. Zhao G. Chang T. Kou K. (2015). Terahertz Spectroscopy of Oligonucleotides in Aqueous Solutions. J. Biomed. Opt. 20 (9), 095009. 10.1117/1.JBO.20.9.095009 26385423

[B30] Tong P. Zhao W.-W. Zhang L. Xu J.-J. Chen H.-Y. (2012). Double-probe Signal Enhancing Strategy for Toxin Aptasensing Based on Rolling Circle Amplification. Biosens. Bioelectron. 33, 146–151. 10.1016/j.bios.2011.12.042 22270050

[B31] Wang F. Zhao D. Dong H. Jiang L. Liu Y. Li S. (2017). Terahertz Spectra of DNA Nucleobase Crystals: A Joint Experimental and Computational Study. Spectrochim. Acta A Mol. Biomol. Spectrosc. 179, 255–260. 10.1016/j.saa.2017.02.037 28273628

[B32] Wang J. Lang T. Hong Z. Xiao M. Yu J. (2021). Design and Fabrication of a Triple-Band Terahertz Metamaterial Absorber. Nanomaterials 11 (5), 1110. 10.3390/nano11051110 33922986 PMC8146610

[B33] Xia L. Cui H.-L. Zhang M. Dang S. Du C. (2019). Broadband Anisotropy in Terahertz Metamaterial with Single-Layer Gap Ring Array. Materials 12 (14), 2255. 10.3390/ma12142255 PMC6678317 31337026

[B34] Xiang Y. Deng K. Xia H. Yao C. Chen Q. Zhang L. (2013). Isothermal Detection of Multiple Point Mutations by a Surface Plasmon Resonance Biosensor with Au Nanoparticles Enhanced Surface-Anchored Rolling Circle Amplification. Biosens. Bioelectron. 49, 442–449. 10.1016/j.bios.2013.04.044 23811476

[B35] Xu W. Xie L. Ying Y. (2017). Mechanisms and Applications of Terahertz Metamaterial Sensing: A Review. Nanoscale 9, 13864–13878. 10.1039/c7nr03824k 28895970

[B36] Yang X. Yang K. Zhao X. Lin Z. Liu Z. Luo S. (2017). Terahertz Spectroscopy for the Isothermal Detection of Bacterial DNA by Magnetic Bead-Based Rolling Circle Amplification. Analyst 142 (24), 4661–4669. 10.1039/c7an01438d 29119154

[B37] Yang Y. Xu D. Zhang W. (2018). High-sensitivity and Label-free Identification of a Transgenic Genome Using a Terahertz Meta-Biosensor. Opt. Express 26 (24), 31589–31598. 10.1364/oe.26.031589 30650742

[B38] Yang K. Li J. Lamy de la Chapelle M. Huang G. Wang Y. Zhang J. (2021). A Terahertz Metamaterial Biosensor for Sensitive Detection of microRNAs Based on Gold-Nanoparticles and Strand Displacement Amplification. Biosens. Bioelectron. 175, 112874. 10.1016/j.bios.2020.112874 33293192

[B39] Yao C. Xiang Y. Deng K. Xia H. Fu W. (2013). Sensitive and Specific HBV Genomic DNA Detection Using RCA-Based QCM Biosensor. Sensors Actuators B Chem. 181, 382–387. 10.1016/j.snb.2013.01.063

[B40] Zhan X. Yang S. Huang G. Yang L. Zhang Y. Tian H. (2021). Streptavidin-functionalized Terahertz Metamaterials for Attomolar Exosomal microRNA Assay in Pancreatic Cancer Based on Duplex-specific Nuclease-Triggered Rolling Circle Amplification. Biosens. Bioelectron. 188, 113314. 10.1016/j.bios.2021.113314 34030095

[B41] Zhang M. Yang Z. Tang M. Wang D. Wang H. Yan S. (2019). Terahertz Spectroscopic Signatures of Microcystin Aptamer Solution Probed with a Microfluidic Chip. Sensors (Basel) 19 (3), 1–12. 10.3390/s19030534 PMC6387113 30696003

[B42] Zheng Q. Xia L. Tang L. Du C. Cui H. (2020). Low Voltage Graphene-Based Amplitude Modulator for High Efficiency Terahertz Modulation. Nanomaterials 10 (3), 585. 10.3390/nano10030585 PMC7153508 32210123

[B21] Zhou J. Zhao X. Huang G. Yang X. Zhang Y. Zhan X. (2021). Molecule-Specific Terahertz Biosensors Based on an Aptamer Hydrogel-Functionalized Metamaterial for Sensitive Assays in Aqueous Environments. ACS Sens. 6 (5), 1884–1890. 10.1021/acssensors.1c00174 33979138

[B27] Zhou R. Wang C. Huang Y. Huang K. Wang Y. Xu W. (2021). Label-free Terahertz Microfluidic Biosensor for Sensitive DNA Detection Using Graphene-Metasurface Hybrid Structures. Biosens. Bioelectron. 188, 113336. 10.1016/j.bios.2021.113336 34022719

