# Supplementary material for: Flexible Terahertz Metamaterial Biosensor for Ultra-Sensitive Detection of Hepatitis B Viral DNA Based on the Metal-Enhanced Sandwich Assay
Source: Front Bioeng Biotechnol. 2022 Aug 5;10:930800. doi: 10.3389/fbioe.2022.930800 (PMC9388765; doi:10.3389/fbioe.2022.930800)
Supplement: Supplementary file 1 [file Datasheet1.docx]

**Supplementary information**

**Flexible Terahertz Metamaterial Biosensor for Ultra-sensitive Detection of Hepatitis B viral DNA based on Metal-Enhanced Sandwich Assay**

Yumin Li^ab#^, Xiaojing Wang^d#^, Yu Liu^b^, Weidong Jin^b^, Huiyan Tian^b^, Xiang Zhao^b^, Fengxin Xie^b^, Xiuming Zhang^a^*, Weiling Fu^b*^ & Yang Zhang^c*^

*^a^ Medical Laboratory of the Third affiliated Hospital of Shenzhen University, Shenzhen, Guangdong 518001, China;*

*^b^ Department of Laboratory Medicine, Southwest Hospital, Third Military Medical University (Army Medical University), Chongqing, 400038，China*

*^c^ Department of Laboratory Medicine, Chongqing University Cancer Hospital, Chongqing, China*

*^d^ Department of Laboratory Medicine, Chifeng Municipal Hospital, Chifeng, Inner Mongolia 024000, China;*

^#^ These authors contributed equally to this work.

* Corresponding Author:

Xiuming Zhang, Email: zxm0760@163.com

Weiling Fu, E-mail: fwl@tmmu.edu.cn

Yang Zhang, Email: [millen001@163.com](mailto:millen001@163.com)


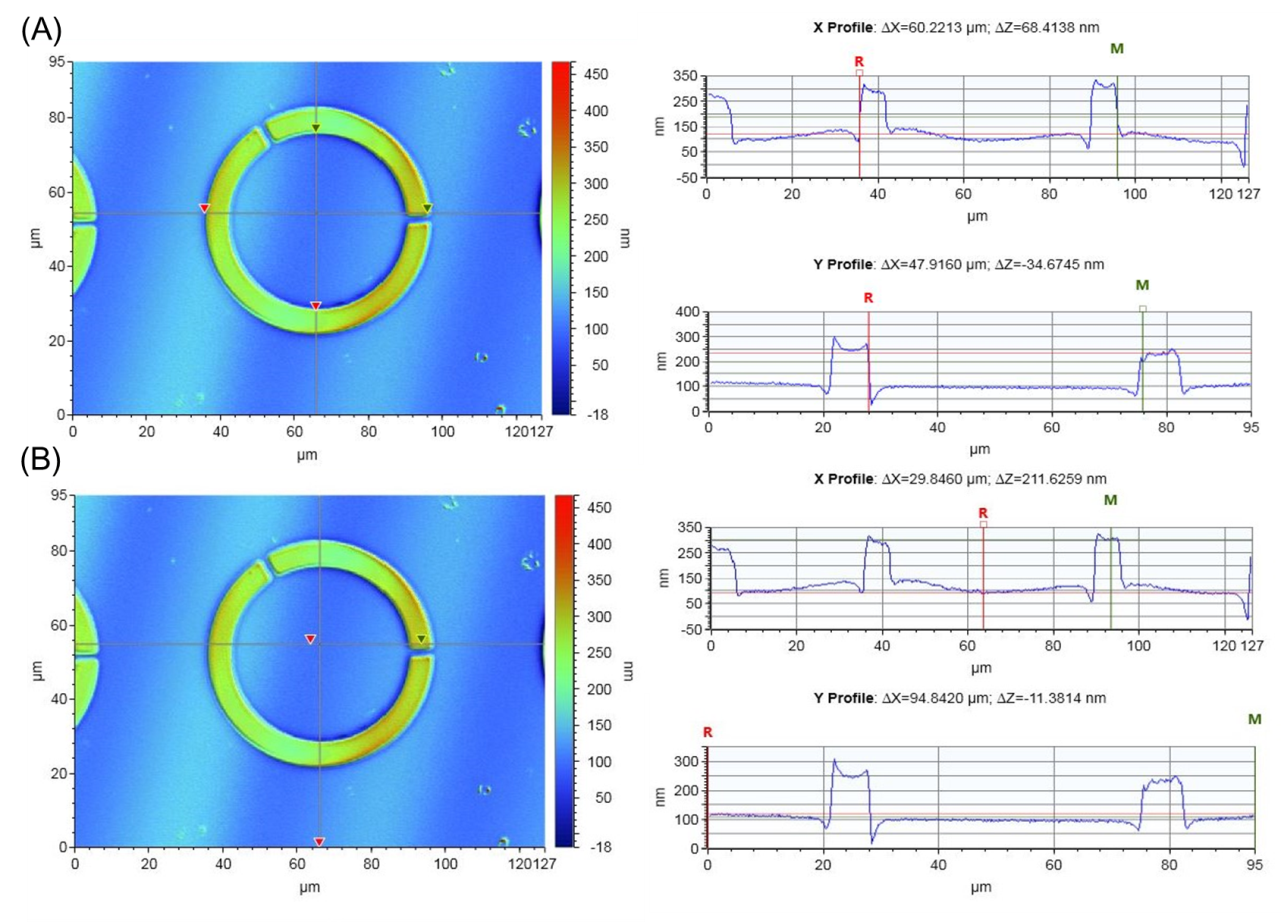


**Fig S1.** Characterization of DSR cell in a square lattice with dimensions of inner and outer diameter (A) and outer radius (B). All dimensions are in μm.


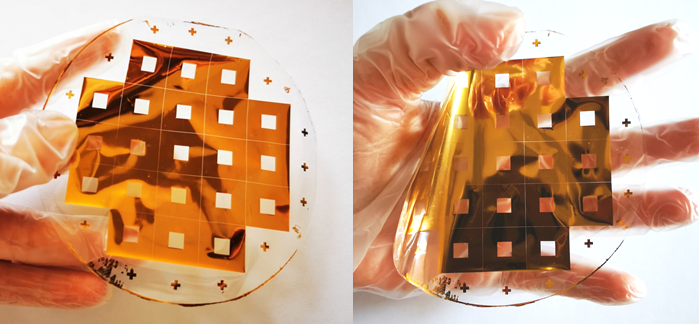


**Fig S2.** Image of the flexible THz metamaterial biochip.


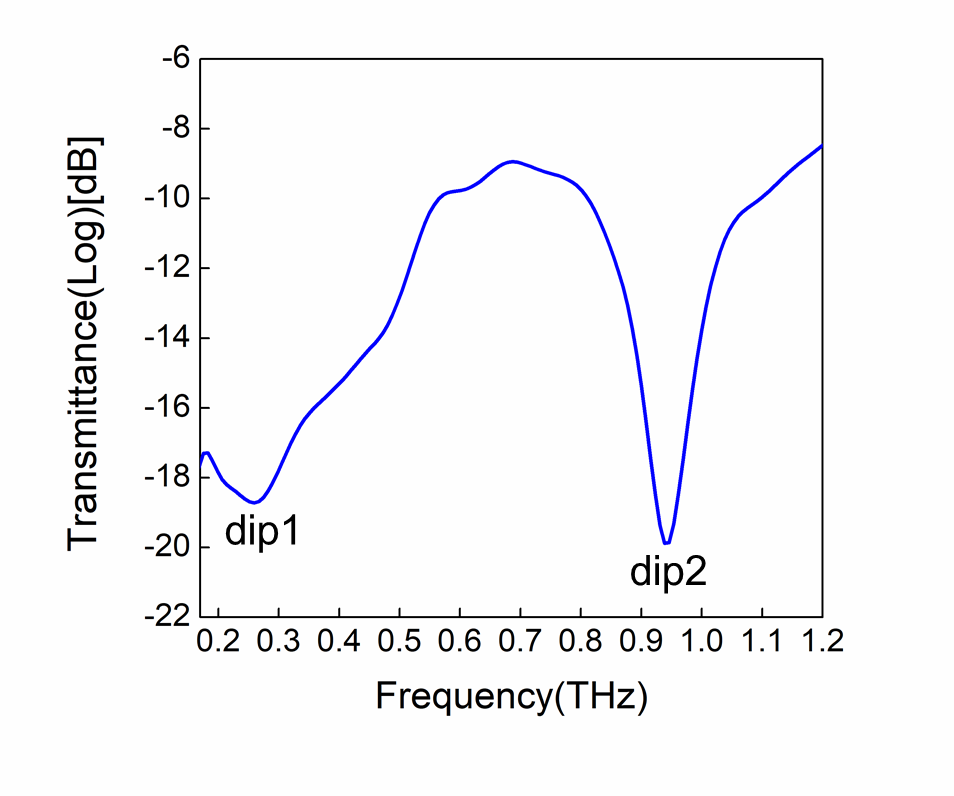


**Fig S3.** THz Transmission spectra of DSRs


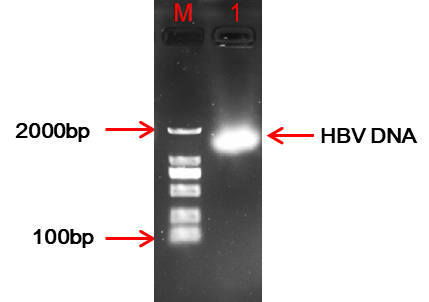


**Fig S4.** Electrophoretic identification of the RCA products of HBV DNA. Lane 1 represent the amplified HBV DNA products.
